# Life cycle of International Congress of Parasitology (ICOPA)

**DOI:** 10.1590/0074-02760190012

**Published:** 2019-05-06

**Authors:** Jokelainen Pikka

**Affiliations:** 1 Statens Serum Institut, Infectious Disease Preparedness, Department of Bacteria, Parasites & Fungi, Laboratory of Parasitology, Copenhagen S, Denmark

**Keywords:** life cycle, parasitology

In August 2018, a high number of parasitologists from all around the world attended the 14th International Congress of Parasitology (ICOPA 2018), in Daegu, Korea.^1^ The organizers of ICOPA 2018 did an excellent job in making the congress a memorable event. When ICOPA 2018 was closing, I had the honor to receive the flag of World Federation of Parasitologists (WFP)^2^ (Figure). The WFP member societies forming the WFP council had voted Copenhagen, Denmark, as the location of ICOPA 2022. With the strong international support, the Scandinavian-Baltic Society for Parasitology (SBSP)^3^ and the Danish Society for Parasitology (DSP)^4^ are looking forward to hosting ICOPAnhagen in August 2022.^5^

This is the life cycle of the ICOPA: prepatent period is four years, patent period is about one week, and transmission is by passing on the WFP flag. All these stages have taken place in different places of the world^1^ ^,^ ^2^ (Table), and detailed observations^6^ and photographic evidence (Figure) have been collected. This life cycle seems to fit ICOPA well, however, the geographic distribution of the locations has not covered all the corners of the world yet.

The hosts of ICOPA are the local and regional parasitological societies. It should be emphasized that ICOPA is a good parasite, it does not harm its hosts. By contrast, the co-existence certainly has symbiotic elements, and hosting an ICOPA is both attractive and competed. In susceptible individuals, ICOPA-infection may cause increased networking and appearing in a bright-colored team T-shirt. It is believed that the hosting leaves a life-long partial immunity ― hosting an ICOPA is typically a once-in-a-lifetime experience.

The host provides the ICOPA all it needs to develop. The host finds a suitable venue for the patent period, welcomes and supports potential new hosts and prepares the transmission ceremony, and works hard to attract a high number of participants to ensure efficient networking and dissemination. It is a joy and a lot of work. The new means of communication, especially social media, are also changing ICOPA. Social media enables networking, visibility, and wide outreach.

Continuation is of course important for the field of parasitology and for ICOPA, and the track record of previous ICOPAs (Table) is convincing. However, it is not easy to think about the next ICOPA, or three or five ICOPAs ahead, while the field suffers from budget cuts and other challenges in many countries. Uncertainty affects research plans both locally and in international collaboration. Moreover, uncertainty may affect potential new hosts that consider hosting an ICOPA. When we started to form the Organizing Committee of ICOPAnhagen, I was a recently-arrived immigrant with a short-term contract, and I actually struggled to have enough money to travel to the Organizing Committee meetings. Diversity in our Organizing Committee was the key to avoid hesitation: the combination of the wisdom and resourcefulness of the more senior colleagues who have experienced more ups and downs, and the innovative new ideas from the early career colleagues. Experience and enthusiasm.

Importantly, the participants of the global congress ICOPA should consist of parasitologists from all over the world. Moreover, the event should inspire new generations of parasitologists to ensure the future of parasitology. There is a challenge: for example, some geographical regions and early-career researchers are often underrepresented. There is room for improvement in both diversity and inclusiveness, and this is something I find important. Earlier I have myself been, twice, in a situation where I did not have the financial means to attend an ICOPA, and I was unsuccesful in finding funding for my students to attend as well. While I did feel ICOPA was a major congress for me and my students, attending required resources I did not have. It is important to ask what creates the feeling of belonging and what are the barriers. The barriers are not equally distributed. For some it is easy to attend, others have to overcome numerous barriers first. Each and every participant is valuable and contributes to the success and impact of the ICOPA.

Each ICOPA is a unique opportunity to discuss and address timely challenges, which may include emerging parasitic diseases, concerns within the community of parasitologists, and challenges of science-to-policy. Meeting peers, in person, can result in life-long collaborations, and sharing experiences and joining forces helps to overcome constraints present in different geographical regions and fields of parasitology. For this, it is of utmost relevance that the different regions, different voices, and various fields of parasitology are present at ICOPA and share their challenges and experiences there.

**Figure:**
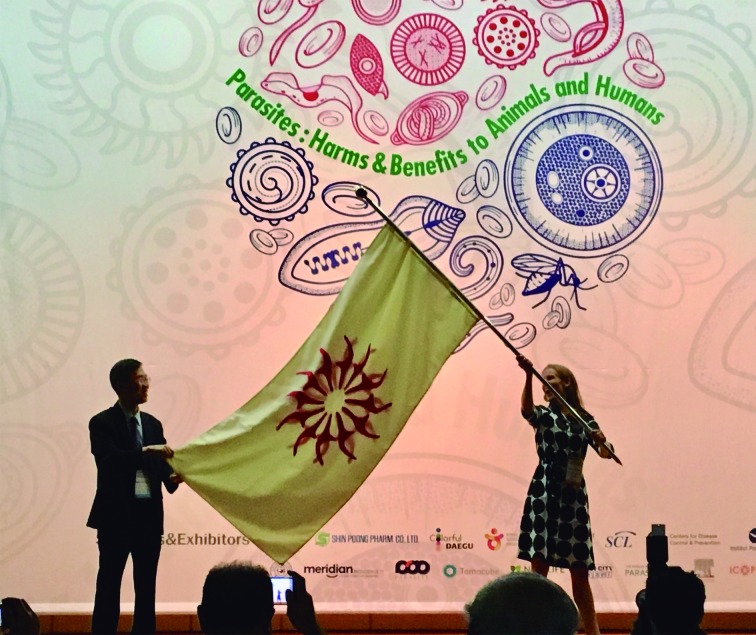
The flag of World Federation of Parasitologists is passed on from the International Congress of Parasitology 2018 (ICOPA 2018) Korean Organizing Committee to the Organizing Committee of ICOPAnhagen, in Daegu, Korea, on August 24, 2018. Photograph by Russell Stothard.

**TABLE:** International Congress of Parasitology (ICOPA) locations over time. Adapted from^(1)^

ICOPA 2022	Copenhagen	Denmark
ICOPA 2018	Daegu	Korea
ICOPA 2014	Mexico City	Mexico
ICOPA 2010	Melbourne	Australia
ICOPA 2006	Glasgow	UK
ICOPA 2002	Vancouver	Canada
ICOPA 1998	Chiba	Japan
ICOPA 1994	Izmir	Turkey
ICOPA 1990	Paris	France
ICOPA 1986	Brisbane	Australia
ICOPA 1982	Toronto	Canada
ICOPA 1978	Warsaw	Poland
ICOPA 1974	Munich	Germany
ICOPA 1970	Washington DC	USA
ICOPA 1964	Rome	Italy

Celebrating achievements and progress is also important. ICOPA, being held every fourth year, is an excellent forum for this ― an observational point in the history of parasitology. This history is being written. Right now is the time to make research plans that will yield interesting results to be presented in 2022, and the work to overcome the relevant barriers needs to start now too. Let us make the best out of the next patent period in 2022, let us make it an event of high scientific quality, diversity and inclusiveness, and impact.
